# Structural insight into the substrate recognition and transport mechanism of the human LAT2–4F2hc complex

**DOI:** 10.1038/s41421-020-00207-4

**Published:** 2020-11-10

**Authors:** Renhong Yan, Jiayao Zhou, Yaning Li, Jianlin Lei, Qiang Zhou

**Affiliations:** 1 Zhejiang Provincial Laboratory of Life Sciences and Biomedicine, Key Laboratory of Structural Biology of Zhejiang Province, School of Life Sciences, Westlake University, 18 Shilongshan Road, Hangzhou, Zhejiang 310024 China; 2 Institute of Biology, Westlake Institute for Advanced Study, 18 Shilongshan Road, Hangzhou, Zhejiang 310024 China; 3 Beijing Advanced Innovation Center for Structural Biology, Tsinghua-Peking Joint Center for Life Sciences, School of Life Sciences, Tsinghua University, Beijing 100084, China; 4 Technology Center for Protein Sciences, Ministry of Education Key Laboratory of Protein Sciences, School of Life Sciences, Tsinghua University, Beijing 100084, China

**Keywords:** Structural biology, Cell biology

Dear Editor,

The l-type amino acid transporters (LATs) mediate neutral amino acids and thyroid hormones across membrane^1–5^. LAT2, which is mainly expressed in kidney and small intestine, has a broader substrate range than LAT1, including small amino acids^6–9^. LAT1 or LAT2 are the light chains of the heterodimeric amino acid transporters (HATs), which are composed of a light chain and a heavy chain. The 4F2 cell-surface antigen heavy chain (4F2hc) is the heavy chain for LAT1 and LAT2, playing roles in plasma membrane localization of LATs^4^ and required for the stability and the transport activity of HATs^10^. The first cryo-EM structures of the human LAT1–4F2hc complex had been solved recently, but the native substrates bound structure of HATs still remains unknown. Here, we report the cryo-EM structures of the human LAT2–4F2hc complex bound with substrate Leu or Trp at resolution of 2.9 or 3.4 Å, respectively. These structures exhibit an inward-open conformation, similar to that of the human LAT1–4F2hc complex. The substrates Leu and Trp are all bound at the bottom of the inner pocket of the transporter, while Trp might adopt two different binding modes. Structural analysis and biochemical assays provide important basis for the working mechanism of HATs, especially by elucidating the substrate-binding mechanism.

The details of recombinant expression and purification of LAT2–4F2hc complex are described in Supplementary Methods and Materials. The complex appeared to be homogeneous after two-step affinity purification (Supplementary Fig. S1a). The purified complex was reconstituted into liposome vesicles and subjected to counterflow assay. The *K*_m_ value is ~177 ± 21.3 μM, which is comparable to previous report in cell-based assays (Supplementary Fig. S1b, c)^6^. The substrates competition assay showed that LAT2–4F2hc can not only transport the large neutral amino acids, such as Phe, Ile, Leu, and Trp, but also the small neutral amino acids such as Ala, Ser, and Thr (Supplementary Fig. S1d).

To investigate the substrates recognition mechanism of HATs, we tried to solve the LAT2–4F2hc structures bound with different substrates. The cryo-EM structures of the LAT2–4F2hc complex incubated with Leu or Trp were determined at 2.9 and 3.4 Å resolution, respectively (Supplementary Figs. S2–S4). The cryo EM maps of LAT2–4F2hc + Leu and LAT2–4F2hc + Trp are very similar to each other (Supplementary Fig. S2i). Nearly all of sequences of both LAT2 and 4F2hc were clearly resolved (Fig. 1a, b and Supplementary Fig. S4), except the N-terminal residues 1–162 for 4F2hc and 1–40 for LAT2, the density of which was invisible in the cryo-EM map.

**Fig. 1:**
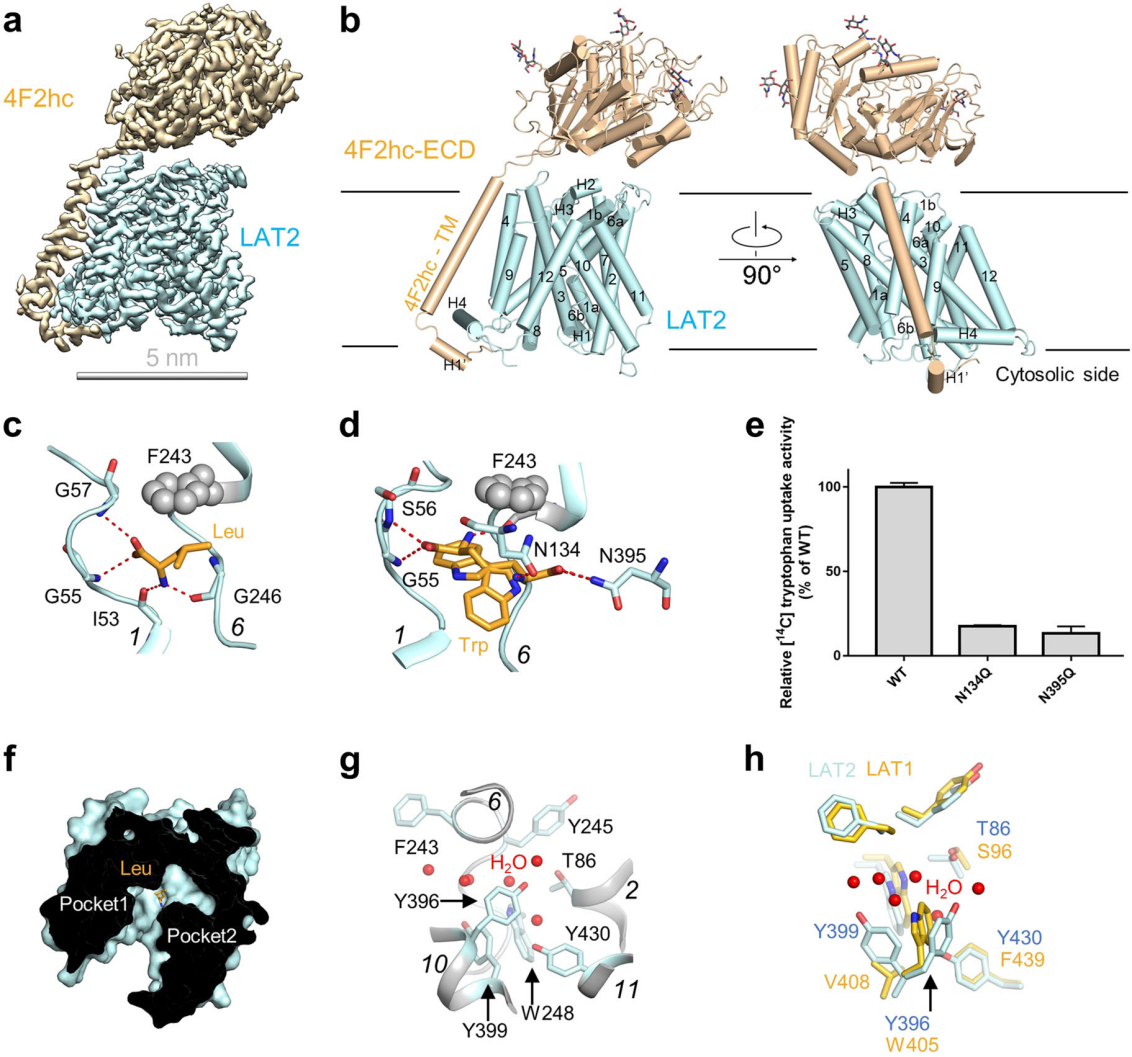
Cryo-EM structure of the human LAT2–4F2hc complex. **a** The overall Cryo-EM map of the human LAT2–4F2hc + Leu complex. LAT2 and 4F2hc are cyan and wheat, respectively. **b** Overall structure of the LAT2–4F2hc + Leu complex. The glycosylation moieties are shown as sticks. H helix, TM transmembrane domain, ECD extracellular domain. **c** The Leu-binding site. **d** The Trp-binding site. Trp substrate might have two binding positions. **e** Mutations of the Trp-binding residues lead to a decreased transport activity. Data are mean ± s.d. of three independent experiments. **f** The two separated pockets in LAT2. The pockets 1 and 2 are separated by the substrate. **g** The water distribution in the pocket 2 of LAT2. **h** The alignment of the second pocket of LAT1 with LAT2. The residues of LAT1 and LAT2 are shown as yellow and cyan, respectively.

The overall structure of LAT2–4F2hc revealed an inward-open conformation, with Leu or Trp substrate binding to the bottom of the inner pocket of LAT2 (Fig. 1c, d and Supplementary Fig. S4c, e). Leu is bound between the unwound region of TM1 and TM6 in a classical mode. The α-carboxylate group of Leu forms two hydrogen bonds with the amide nitrogen atoms of Gly55 and Gly57 in TM1, while the α-amino group of Leu forms hydrogen bonds with the carbonyl oxygen atoms of Ile53 in TM1 and Gly246 in TM6. The side chain of Leu is stabilized by the benzene ring of Phe243. The binding mode shown here is quite similar to the BCH bound in LAT1, implicating a common substrate recognition mechanism shared by LAT1 and LAT2^10^. Interestingly, the Trp substrate shows a triangular star-shaped density in the map (Supplementary Fig. S4d, e), into which we assigned two binding positions for Trp. In position 1, Trp exhibits similar binding mode to Leu, while in position 2, the α-amino and α-carboxylate group of Trp mainly interact with the TM3 and TM10 in LAT2, forming hydrogen bonds with Asn134 in TM3 and Asn395 in TM10, respectively. To validate the binding modes, we introduced the N134Q, F243A, and N395Q mutants in LAT2 and set up the counterflow assay. Results showed that the F243A mutation had almost no transport activity (Supplementary Fig. S5b, c). The N134Q and N395Q mutations remained ~20% transport activity compared with wild type (WT) in the [^14^C]-Trp and Leu counterflow assay (Fig. 1e), or remained ~15% and ~40% transport activity in the [^3^H]-Leu and Leu counterflow assay (Supplementary Fig. S5a), respectively, suggesting their important roles in the transport cycle of LAT2.

The putative dual-binding-mode does not exclude Leu, as the N134Q and N395Q mutations affect the LAT2-mediated transport of both Trp and Leu. The cryo EM density is not observed for the position 2 of Leu as the smaller size of Leu and the potential weaker interactions between the side chain of Leu and the gating residue Phe243 of LAT2. We propose that the Asn134-binding and Asn395-binding state might represent an intermediate state of substrate recognition and transport during an alternating assess cycle. Actually, the corresponding residues for Asn134 and Asn395 in LAT2 are Ser and Asn in LAT1 and ASC1 (Supplementary Fig. S6), which are capable to form hydrogen bonds with substrates, suggesting the putative dual-binding-mode described here might also exist in LAT1 and ASC1.

In the LAT2 structure, there is a remote pocket near the classical substrate-binding pocket. We called these two pockets as pocket 1 and 2 for simplicity (Fig. 1f). The pocket 1 and 2 are separated by the substrate Leu or Trp, while they are combined together in LAT1^10^. On account of the high-resolution structure of LAT2–4F2hc+Leu, we assigned six water molecules in pocket 2, indicating the solvent accessibility for the pocket 2 (Fig. 1g). Structural comparison and sequence alignment between LAT1 and LAT2 shows that the major different residues in pocket 2 are Thr86 (Ser96 in LAT1), Tyr396 (Trp405 in LAT1), Tyr399 (Val408 in LAT1), and Tyr430 (Phe439 in LAT1) (Fig. 1h and Supplementary Fig. S6). The substitution to bigger residue in LAT2 at these sites (except the substitution from Trp405 of LAT1 to Tyr396 of LAT2) may facilitate LAT2 to bind smaller substrates. Interestingly, sequence alignment of HAT members (Supplementary Fig. S6) also shows that ASC1, a member of the SLC7 family that mediates the transport of the small amino acids (such as alanine, serine, and cysteine), has the same residues as LAT2 at these sites (Thr86, Tyr396, Tyr399, and Tyr430 of ASC1). Mutation of the residues in pocket 2 to Ala led to markedly decreased transport activity compared with WT complex, indicating the important role of pocket 2 in transport cycle (Supplementary Fig. S5b, c). Since the pocket 1 is quite conserved in the LeuT-fold transporter from prokaryotes to eukaryotes, the identification of pocket 2 in LAT2–4F2hc might help understand the transport mechanism of HATs. To investigate whether the pocket 2 determines the substrate selectivity for LAT1 and LAT2, we introduced triple substitution mutation (Y396W/Y399V/Y430F, named as 3X) in pocket 2 of LAT2 to mimic LAT1. Since LAT2 can transport Ser while LAT1 cannot do this^10^, we added 1 mM Ser to see whether this can affect the transport activity of the 3X mutant or not. Results showed that 3X mutant remained ~45% transport activity and it can still transport Ser substrate (Supplementary Fig. S5d), indicating this 3X mutant might not fully mimic LAT1.

We also analyzed the substrate specificity of HAT members. Among the light chain proteins of HATs, only b^0,+^AT, y^+^LAT1, y^+^LAT2 can mediate the transmembrane transport of dibasic amino acids. These three proteins contain acidic residues in pocket 1 region (Asp233 of b^0,+^AT, Asp243 of y^+^LAT1, Asp251 of y^+^LAT2), corresponding to which LAT1 and LAT2 have non-charged residues (Gly255 and Asn258 for LAT1, Gly246 and Asn249 for LAT2). The charged residues in pocket 1 might interact with the charged side chain of substrates to facilitate the transport cycle. This may explain why LAT1 and LAT2 are specific to neutral amino acids.

In summary, the cryo-EM structures and structure-guided mutational analysis reported in this work provide clues into the substrates recognition mechanism of eukaryotic HAT antiporters and give hints for the further drug design of related diseases.

## Supplementary information

Supplementary Information

## Data Availability

Atomic coordinates and cryo EM density maps of the LAT2–4F2hc complex in complex with tryptophan (PDB: 7CMH; EMDB: EMD-30406) or leucine (PDB: 7CMI; EMDB: EMD-30407) have been deposited in the Protein Data Bank (http://www.rcsb.org) and the Electron Microscopy Data Bank (https://www.ebi.ac.uk/pdbe/emdb/). Correspondence and requests for materials should be addressed to Q. Z. (zhouqiang@westlake.edu.cn).

## Supplementary information

**Supplementary Information** accompanies the paper at (10.1038/s41421-020-00207-4).
