# Supplementary material for: Structural insight into the substrate recognition and transport mechanism of the human LAT2–4F2hc complex
Source: Cell Discov. 2020 Nov 10;6:82. doi: 10.1038/s41421-020-00207-4 (PMC7653941; doi:10.1038/s41421-020-00207-4)
Supplement: Supplementary file 1 — Supplementary Information [file 41421_2020_207_MOESM1_ESM.pdf]

Supplementary information

Methods and Materials

Protein preparation

The full-length human cDNA of LAT2 (accession number: NM_012244.4) was subcloned into pCAG with N-terminal FLAG tag and 4F2hc (isoform b, accession number: NM_001012662.2) into pCAG with N-terminal 10×His tag. The mutations were generated by a standard two-step PCR.

For protein expression, LAT2 and 4F2hc were co-expressed in HEK 293F cells (Invitrogen) cultured in SMM 293T-I medium (Sino Biological Inc.) at 37 °C under 5% CO_2 in a Multitron-Pro shaker (Infors, 130 rpm). For transfection, 50 ml mixture containing 3 mg of polyethylenimines (PEIs) (Polysciences), 0.75 mg of the LAT2 plasmid, 0.75 mg of the 4F2hc plasmid and fresh medium was added into a liter of cell culture whose cell density reached 2.0×10^6/ml. 48-60 hours after transfection cells were harvested by centrifugation at 3800×g for 10 mins and resuspended in a buffer containing 25 mM Tris (pH 8.0), 150 mM NaCl, and mixture of three protease inhibitors, aprotinin (1.3 μg/ml, AMRESCO), pepstatin (0.7 μg/ml, AMRESCO) and leupeptin (5 μg/ml, AMRESCO).

For incubation of the complex sample with tryptophan or leucine, after incubating at 4 °C for 2 hours with 1% GDN (w/v) (Anatrace), cells was centrifugated at 18,700×g for 45 mins to remove cell debris. The supernatant was loaded onto anti-FLAG M2 affinity resin (Sigma). The resin was washed with the wash buffer containing 25 mM Tris (pH 8.0), 150 mM NaCl, 0.05% GDN (w/v), following by protein eluted with wash buffer plus 0.2 mg/mL FLAG peptide. Then elution of anti-FLAG M2 affinity resin was further purified with Ni-NTA affinity resin (Qiagen). Wash buffer and elution buffer of nickel resin was wash buffer mentioned above plus 10 mM and 300 mM imidazole respectively. Then the protein complex was subjected to size-exclusion chromatography (Superose 6 Increase 10/300 GL, GE Healthcare) in buffer containing 25 mM Tris (pH 8.0), 150 mM NaCl and 0.02% GDN. The peak fractions

were collected and concentrated for EM analysis.

For transport activity assay, the purification procedure of protein complex was almost same as that was described above except the membrane fraction was solubilized at 4 °C for 2 hours with 1% (w/v) LMNG (Anatrace) supplemented with 0.1% (w/v) cholesteryl hemisuccinate Tris salt (Anatrace) instead of GDN. The peak fractions were collected and stored at -80℃ for proteoliposomes preparation.

In vitro transport activity assay

Liposomes and proteoliposomes were prepared as described previously with a slight modification [1]. The reaction buffer inside liposomes and proteoliposomes contains 20 mM potassium phosphate (pH 6.5), 150 mM KCl and 10 mM L-leucine (Sigma). All transport activity assays were performed at room temperature. The reaction was started by adding 100 μL of reaction buffer containing 20 mM potassium phosphate (pH 6.5), 150 mM KCl and 0.1 μM (1 μCi) L-[^{3}H] Leucine or 7.72 μM (0.04 μCi) L-[^{14}C] Tryptophan (PerkinElmer Life Sciences) to 4 μL of proteoliposome. L-[^{3}H] Leucine or L-[^{14}C] Tryptophan uptake was stopped after 1 min or 5 mins by rapidly filtering the reaction solution through a 0.22 μm GSTF filter (Millipore) and washed with 2 mL of ice-cold wash buffer (20 mM potassium phosphate (pH 6.5) and 150 mM KCl). The filter was then used for liquid scintillation counting.

To initiate the substrate competition assay, extra 1mM unlabeled amino acids was added into reaction buffer. The measurement of K_m and V_{max} was performed in the presence of unlabeled leucine at the indicated concentrations in reaction buffer outside the proteoliposome, and the uptake of ^{3}H-labeled leucine was stopped after 15 s. In each measurement, liposome containing no protein was involved in as empty control. Data was processed by GraphPad Prism software.

Cryo-EM sample preparation and data acquisition

The purified LAT2-4F2hc complex was concentrated to ~ 10 mg/mL and incubated

with 10 mM Leu or 2 mM Trp for 2 hours if necessary before being applied to the grids. Aliquots (3.5 μL) of the protein complex were placed on glow-discharged holey carbon grids (Quantifoil Cu R1.2/1.3). The grids were blotted for 3s or 3.5 s and flash-frozen in liquid ethane cooled by liquid nitrogen with Vitrobot (Mark IV, Thermo Fisher Scientific). The prepared grids were transferred to a Titan Krios operating at 300 kV equipped with Gatan K3 Summit detector and GIF Quantum energy filter for the LAT2-4F2hc+Leu complex, or with Cs corrector, Gatan K2 Summit detector and GIF Quantum energy filter for the LAT2-4F2hc+Trp complex. A total of 3,384 and 5,030 movie stacks were automatically collected using AutoEMation for LAT2-4F2hc+Leu and LAT2-4F2hc+Trp, respectively, with a slit width of 20 eV on the energy filter and a preset defocus range from -1.2 μm to -2.2 μm in super-resolution mode. The total electron dose was approximately 48 $e^-/Å^2$ for each stack, which contained 32 frames. The stacks were motion corrected and dose-weighted [2] with MotionCor2 [3] and binned 2-fold, resulting in a pixel size of 1.087 Å/pixel or 1.091 Å/pixel for the LAT2-4F2hc+Leu or the LAT2-4F2hc+Trp complex, respectively. The defocus values were estimated with Gctf [4].

Data processing

A total of 2,830,087 or 3,217,534 particles were automatically picked from 2,357 or 4,656 manually selected micrographs using Relion[5-10] for the LAT2-4F2hc+Leu or LAT2-4F2hc+Trp complex, respectively. After 2D classification, a total of 1,584,241 or 2,569,261particles were selected for the LAT2-4F2hc+Leu and LAT2-4F2hc+Trp complex, respectively. The selected particles were subjected to global angular searching 3D classification against an initial model generated with Relion. For each of the last several iterations of the global angular searching 3D classification, a local angular searching 3D classification was performed, during which the particles were classified into 4 classes. A total of 1,584,241 or 1,152,978 non-redundant good particles were selected from the local angular searching 3D classification for the LAT2-4F2hc+Leu and LAT2-4F2hc+Trp complex, respectively. Then, these selected particles were subjected to multi-reference 3D classification and local defocus

refinement. The overall resolutions of the 3D auto-refinement after post-processing were 2.9 Å or 3.4 Å with a particle number of 751,924 or 713,248, for the LAT2-4F2hc+Leu and LAT2-4F2hc+Trp complex, respectively.

The 2D classification, 3D classification and auto-refinement were performed with Relion 3. The local defocus refinement was accomplished with Gctf. The resolution was estimated with the gold-standard Fourier shell correlation 0.143 criterion [11,12] with high-resolution noise substitution [13].

Model building and structure refinement

Model building of the LAT2-4F2hc+Trp and the LAT2-4F2hc+Leu complex was performed with Coot[14] based on the cryo-EM maps with the PDB model of the LAT1-4F2hc complex (PDB ID: 6IRT) as a starting template. The subsequent modeling was performed in Coot with aromatic residues as land markers, as most of these residues were clearly visible in our cryo-EM maps. Each residue was manually checked with the chemical properties considered during model building.

A total of 927 amino acid residues were constructed for the LAT2-4F2hc+Trp and the LAT2-4F2hc+Leu complex. The N-terminal sequences of both 4F2hc and LAT2 were not modeled because of the invisibility of the corresponding density in the map. 8 sugar moieties, 2 lipid moieties, and 1 ligand amino acid molecule were assigned for LAT2-4F2hc complex according to the cryo EM maps. There were 6 water molecules assigned for the LAT2-4F2hc+Leu complex.

Structure refinement was performed with Phenix [15] with secondary structure and geometry restraints to prevent structure overfitting. To monitor the overfitting of the model, the model was refined against one of the two independent half maps from the gold-standard 3D refinement approach. Then, the refined model was tested against the other map [16]. Statistics associated with data collection, 3D reconstruction and model refinement can be found in Supplemental Data Table S1.

References

1 R. Yan, X. Zhao, J. Lei, Q. Zhou, Structure of the human LAT1-4F2hc heteromeric amino

acid transporter complex. Nature 568, 127-130 (2019).
2 T. Grant, N. Grigorieff, Measuring the optimal exposure for single particle cryo-EM using a 2.6 A reconstruction of rotavirus VP6. *eLife* **4**, e06980 (2015).
3 S. Q. Zheng *et al.*, MotionCor2: anisotropic correction of beam-induced motion for improved cryo-electron microscopy. *Nature methods* **14**, 331-332 (2017).
4 K. Zhang, Gctf: Real-time CTF determination and correction. *Journal of structural biology* **193**, 1-12 (2016).
5 S. H. Scheres, A Bayesian view on cryo-EM structure determination. *Journal of molecular biology* **415**, 406-418 (2012).
6 S. H. Scheres, RELION: implementation of a Bayesian approach to cryo-EM structure determination. *Journal of structural biology* **180**, 519-530 (2012).
7 S. H. Scheres, Semi-automated selection of cryo-EM particles in RELION-1.3. *Journal of structural biology* **189**, 114-122 (2015).
8 D. Kimanius, B. O. Forsberg, S. H. Scheres, E. Lindahl, Accelerated cryo-EM structure determination with parallelisation using GPUs in RELION-2. *eLife* **5**, (2016).
9 J. Zivanov *et al.*, New tools for automated high-resolution cryo-EM structure determination in RELION-3. *eLife* **7**, (2018).
10 A. Punjani, J. L. Rubinstein, D. J. Fleet, M. A. Brubaker, cryoSPARC: algorithms for rapid unsupervised cryo-EM structure determination. *Nature methods* **14**, 290-296 (2017).
11 P. B. Rosenthal, R. Henderson, Optimal determination of particle orientation, absolute hand, and contrast loss in single-particle electron cryomicroscopy. *Journal of molecular biology* **333**, 721-745 (2003).
12 S. H. Scheres, S. Chen, in *Nature methods*. (United States, 2012), vol. 9, pp. 853-854.
13 S. Chen *et al.*, High-resolution noise substitution to measure overfitting and validate resolution in 3D structure determination by single particle electron cryomicroscopy. *Ultramicroscopy* **135**, 24-35 (2013).
14 P. Emsley, B. Lohkamp, W. G. Scott, K. Cowtan, Features and development of Coot. *Acta Crystallogr D Biol Crystallogr* **66**, 486-501 (2010).
15 P. D. Adams *et al.*, PHENIX: a comprehensive Python-based system for macromolecular structure solution. *Acta Crystallogr D Biol Crystallogr* **66**, 213-221 (2010).
16 A. Amunts *et al.*, Structure of the yeast mitochondrial large ribosomal subunit. *Science (New York, N.Y.)* **343**, 1485-1489 (2014).

Supplementary information, Figure S1

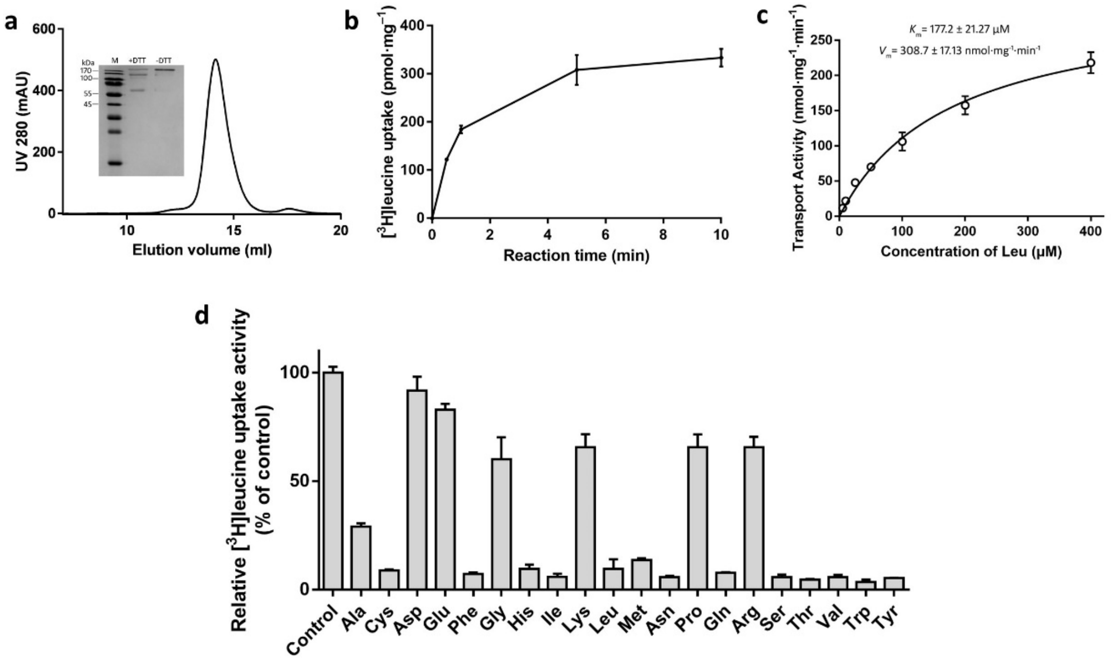

Figure S1 Biochemical Characterizations of human LAT2-4F2hc complex. a Representative SEC purification of the LAT2–4F2hc complex. The protein complex was extracted in the presence of GDN. Inset, SDS–PAGE under reducing (+dithiothreitol (DTT)) or oxidizing (−DTT) conditions, visualized by Coomassie blue staining. **b** Liposome-based counterflow assay for the LAT2-4F2hc complex. A typical time course of [^{3}H]-leucine transport is shown. **c** *K*m and *V*max values for the wild-type LAT2-4F2hc complex. **d** Competition assay for Leu transport by the wild-type complex.

Supplementary information, Figure S2

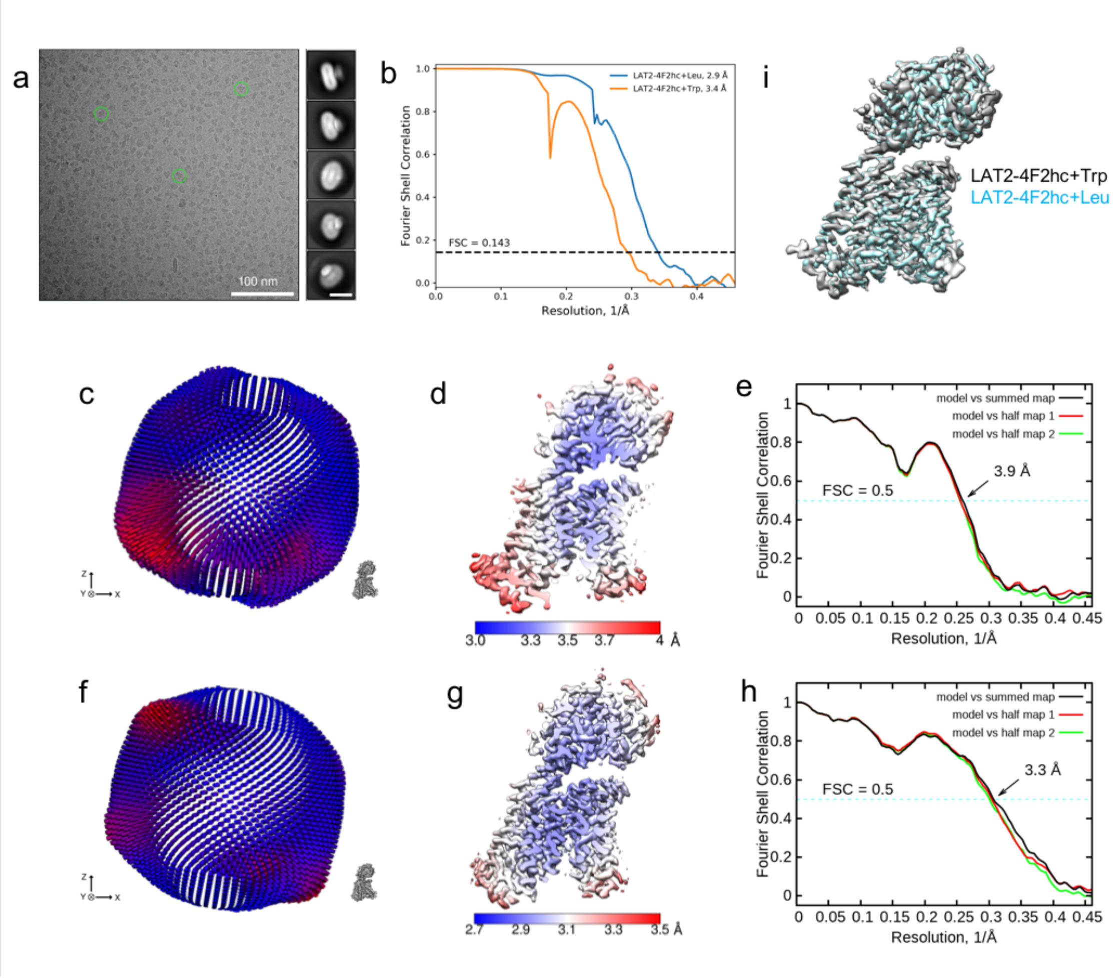

Figure S2 Cryo-EM analysis of the LAT2-4F2hc complex. **a** Representative cryo-EM micrograph and 2D class averages. The scale bar in 2D class averages is 10 nm. **b** Gold standard FSC curve. **c** Euler angle distribution and **d** Local resolution map for the 3D reconstruction of the LAT2-4F2hc+Trp complex. **e** FSC curve of the refined model of the LAT2-4F2hc+Trp complex versus the overall cryo-EM map that it was refined against (black); of the model refined against the first half map versus the same map (red); and of the model refined against the first half map versus the second half map (green). The small difference between the red and green curves indicates that the refinement of the atomic coordinates did not suffer from overfitting. (**f**), (**g**) and (**h**) are same to (**c**), (**d**) and (**e**), respectively, but for the LAT2-4F2hc+Leu complex. **i** The cryo-EM maps of the LAT2-4F2hc+Trp complex and the LAT2-4F2hc+Leu complex are very similar to each other.

Supplementary information, Figure S3

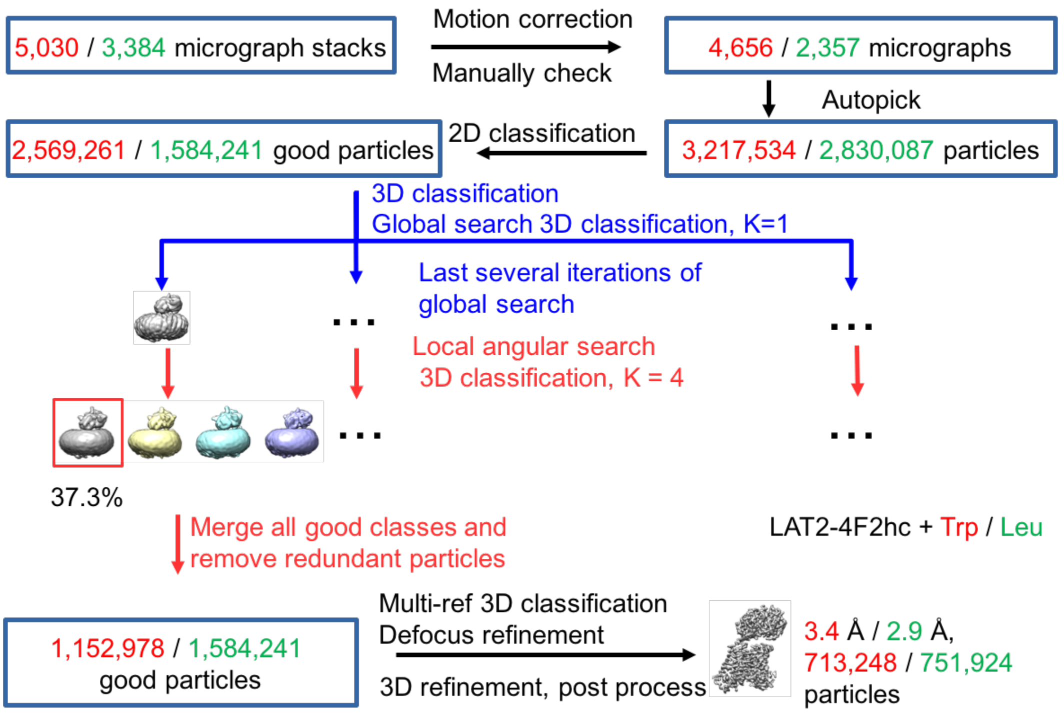

Figure S3 Flowchart for cryo-EM data processing of the human LAT2-4F2hc complex. For details, see 'Data processing' in the Methods section. K, number of classifications.

Supplementary information, Figure S4

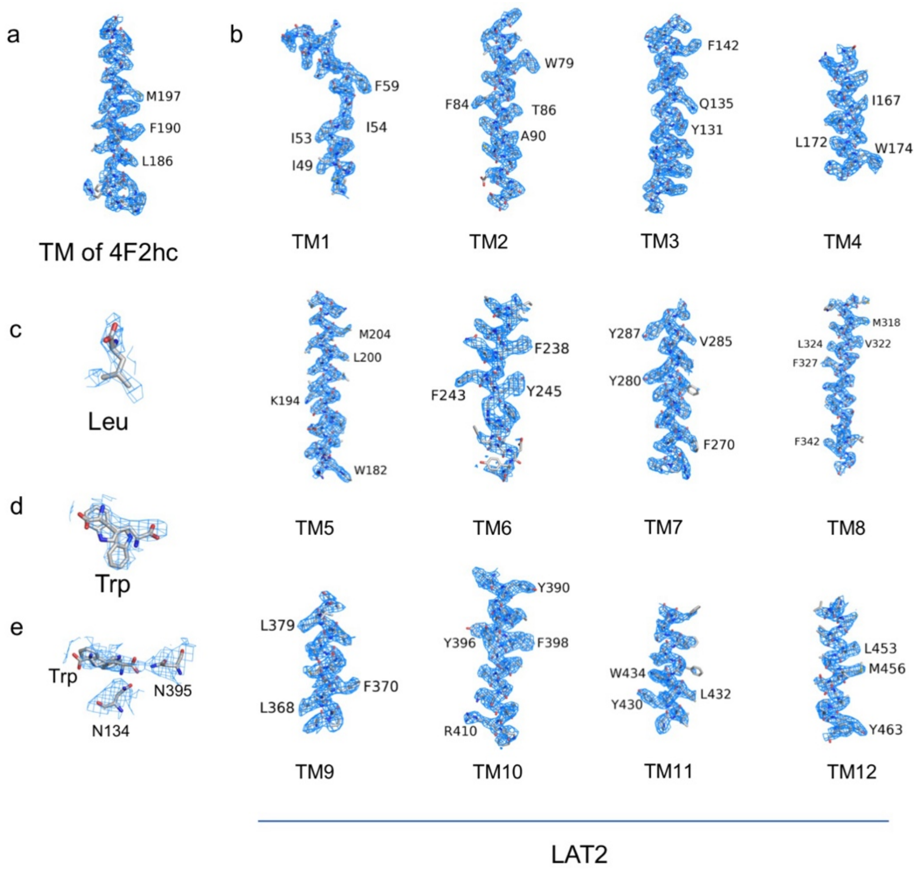

Figure S4 Cryo EM maps of representative segments. **a** The density for the transmembrane helix of 4F2hc in the LAT2-4F2hc+Leu complex. **b** The densities of the 12 TM segments of LAT2 in the LAT2-4F2hc+Leu complex. **c** The Leu density in the cryo EM map of LAT2-4F2hc+Leu complex. **d** The Trp density in the cryo EM map LAT2-4F2hc+Trp complex. **e** is same to d, but in another view to show the surrounding residues Asn134 and Asn395. The threshold is 8 σ for a and b or 4 σ for c, d and e.

Supplementary information, Figure S5

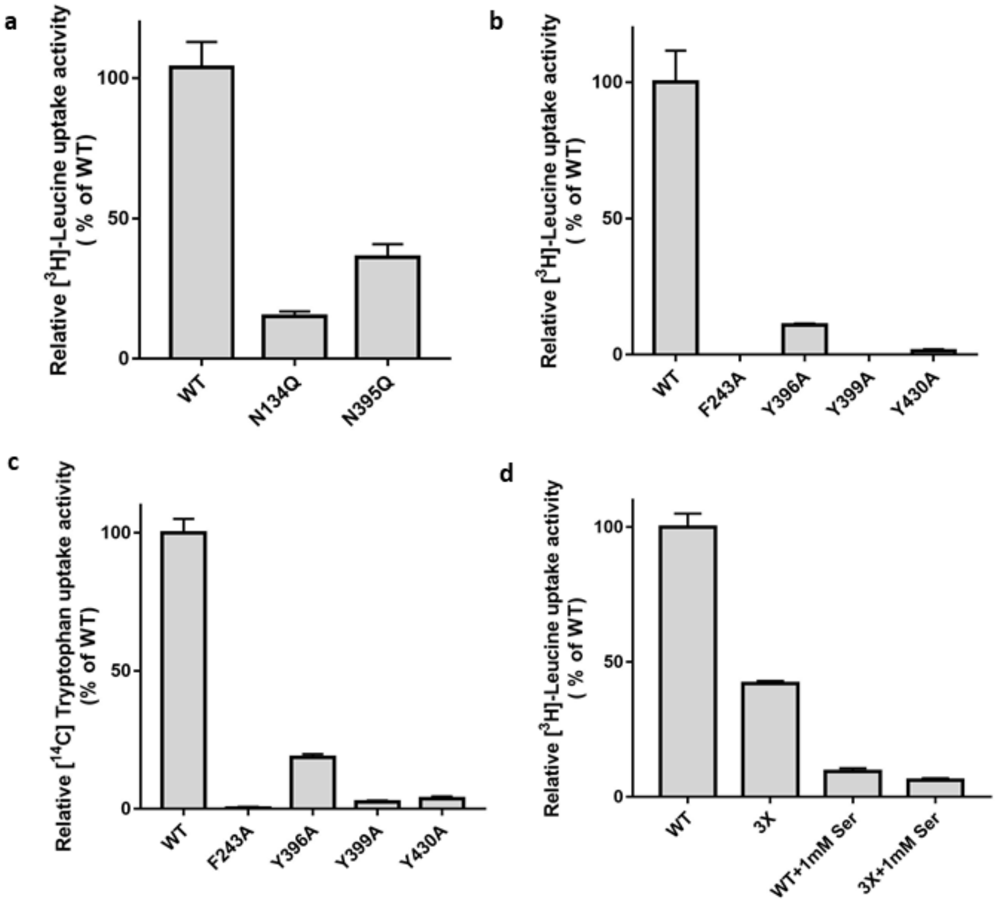

Figure S5. The transport activity of residues involved in pocket 2. **a** The [^{3}H]-Leucine uptake was measured for N134Q or N395Q of LAT1. **b** The [^{3}H]-Leucine uptake was measured for different variants. **c** The The [^{14}C]-Tryptophan uptake was measured for different variants. **d** The [^{3}H]-Leucine uptake was measured for WT and 3X mutant. 1mM Ser was added to compete [^{3}H]-Leucine uptake. Data are mean ± s.d. of three independent experiments.

Supplementary information, Figure S6

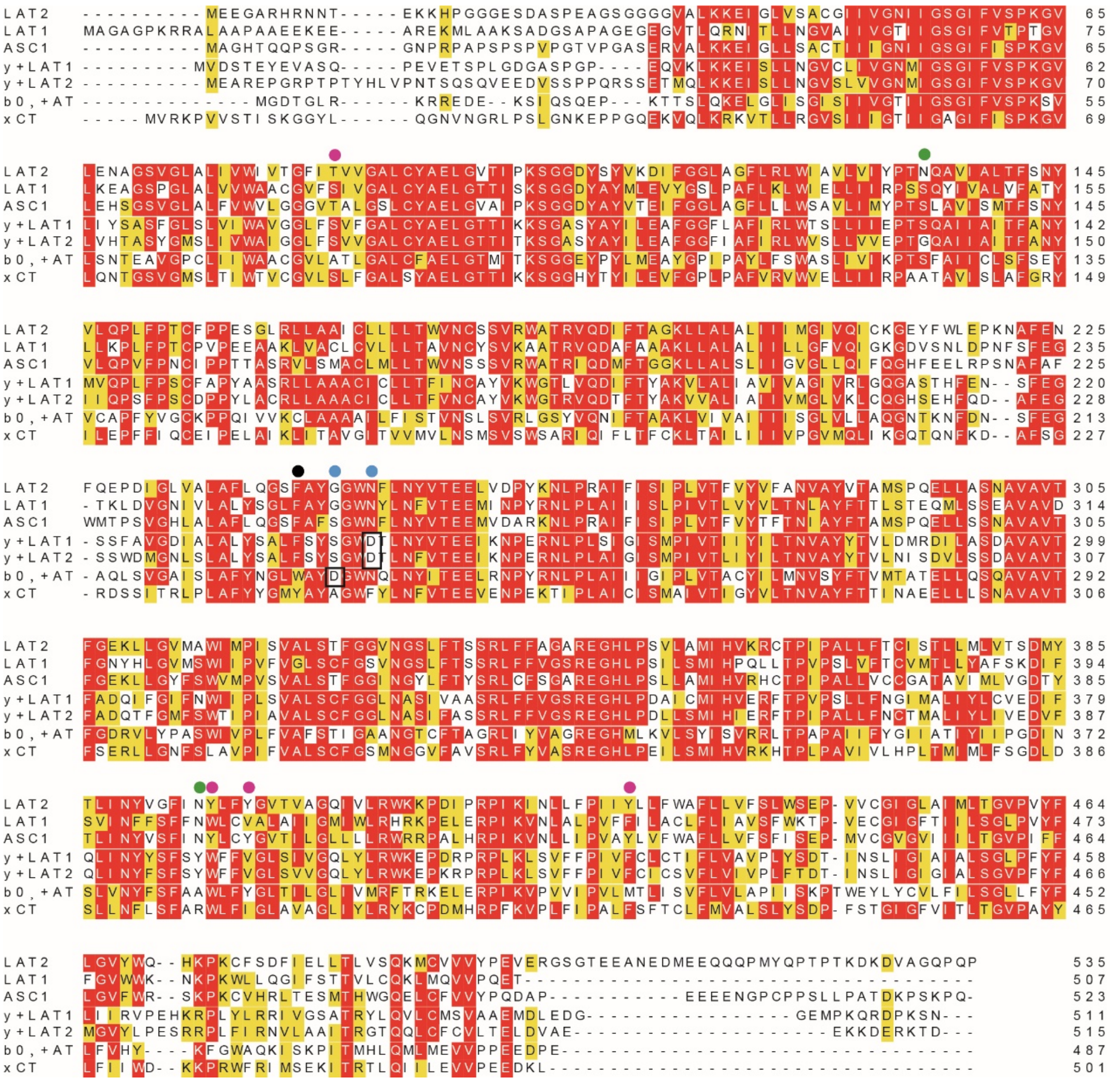

Figure S6. Sequence alignment of LAT2 homologues. The sequences were aligned using clustalX. The seven aligned sequences are LAT2, LAT1, ASC1, y^+LAT1, y^+LAT2, $b^{0,+}$AT, xCT from Homo sapiens. Amino acids that are identical or conserved in at least four sequences are coloured red or yellow, respectively. The gating residue Phe243 is labelled with a solid black circle. The residues that are involved in substrate specificity of neutral and smaller amino acids are marked with solid blue and magenta circles, respectively. Asp of $b^{0,+}$AT, y^+LAT1 and y^+LAT2 corresponding to which LAT1 and LAT2 have non-charged residues are outlined with a black box. The resides important for the second substrate binding mode of LAT2 are indicated by solid green circles. The UNIPROT IDs of aligned sequences are listed as below. LAT2: Q9UHI5; LAT1: Q01650; ASC1: Q9NS82; y^+LAT1: Q9UM01; y^+LAT2: Q92536; $b^{0,+}$AT: P82251; xCT: Q9UPY5.

Supplementary information, Table S1

Table S1 | Data collection, 3D reconstruction and model statistic

**Data collection**		
	LAT2-4F2hc+Trp	LAT2-4F2hc+Leu
EM equipment	Titan Krios (Thermo Fisher Scientific)	
Voltage (kV)	300	
Detector	Gatan K2 Summit	Gatan K3
Energy filter	Gatan GIF Quantum, 20 eV slit	
Pixel size (Å)	1.091	1.087
Electron dose ($e^-/Å^2$)	48	
Defocus range (μm)	-1.2 ~ -2.2	
Number of collected micrographs	5,030	3,384
**3D Reconstruction**		
Software	Relion 3.0	
Symmetry	C1	
Number of used particles	713,248	751,924
Resolution (Å)	3.4	2.9
Map sharpening B-factor ($Å^2$)	-200	-150
**Refinement**		
Software	Phenix	
Cell dimensions		
a=b=c (Å)	279.3	278.3
α=β=γ (˚)	90	
Model composition		
Protein residues	927	
Side chains assigned	911	
R.m.s deviations		
Bonds length (Å)	0.004	0.005
Bonds Angle (˚)	0.955	0.923
Ramachandran plot statistics (%)		
Preferred	92.09	92.63
Allowed	7.69	7.15
Outlier	0.22	0.22
